# Cytokine responses in birds challenged with the human food-borne pathogen *Campylobacter jejuni* implies a Th17 response

**DOI:** 10.1098/rsos.150541

**Published:** 2016-03-16

**Authors:** William D. K. Reid, Andrew J. Close, Suzanne Humphrey, Gemma Chaloner, Lizeth Lacharme-Lora, Lisa Rothwell, Pete Kaiser, Nicola J. Williams, Tom J. Humphrey, Paul Wigley, Stephen P. Rushton

**Affiliations:** 1 School of Biology, Newcastle University, Ridley Building, Newcastle upon Tyne NE1 7RU, UK; 2 Department of Infection Biology, Institute of Global Health, University of Liverpool, Leahurst Campus, Neston, Chester CH64 7TE, UK; 3 Department of Epidemiology and Population Health, Institute of Global Health, University of Liverpool, Leahurst Campus, Neston, Chester CH64 7TE, UK; 4 The Roslin Institute and Royal (Dick) School of Veterinary Science, University of Edinburgh, Midlothian EH25 9RG, UK; 5 College of Medicine, Swansea University, Singleton Park, Swansea SA2 8PP, UK

**Keywords:** gastrointestinal tract, Th1 and Th17 response, structural equation model, bacterial pathogen, gamma-delta T lymphocytes, inflammation

## Abstract

Development of process orientated understanding of cytokine interactions within the gastrointestinal tract during an immune response to pathogens requires experimentation and statistical modelling. The immune response against pathogen challenge depends on the specific threat to the host. Here, we show that broiler chickens mount a breed-dependent immune response to *Campylobacter jejuni* infection in the caeca by analysing experimental data using frequentist and Bayesian structural equation models (SEM). SEM provides a framework by which cytokine interdependencies, based on prior knowledge, can be tested. In both breeds important cytokines including pro-inflammatory interleukin (IL)-1β, , IL-4, IL-17A, interferon (IFN)-γ and anti-inflammatory IL-10 and transforming growth factor (TGF)-β4 were expressed post-challenge. The SEM revealed a putative regulatory pathway illustrating a T helper (Th)17 response and regulation of IL-10, which is breed-dependent. The prominence of the Th17 pathway indicates the cytokine response aims to limit the invasion or colonization of an extracellular bacterial pathogen but the time-dependent nature of the response differs between breeds.

## 1. Introduction

The epithelial lining of the gastrointestinal tract is a high-risk area where pathogens, especially bacteria, can infect a host. On recognition of a pathogen the host immune response involves a number of cells (e.g. natural killer, helper T (Th) cells) and signalling molecules resulting in a complex network of interactions involving the innate and adaptive immune systems [1,2]. A key component of this immune network is cytokines [3], which help control and regulate the host's immune system. Cytokine activation initiates a cascade of responses dependent on the initial stimuli that can regulate innate and adaptive immune systems [2,4–6]. The cytokine micro-environment during Th-cell activation determines effector T-cell differentiation through selective signal transducer and activator of transcription (STAT) proteins, guided by key transcription factors*.* Immunopathogenesis, disease progression and development of auto-immune diseases require an understanding of how cytokines interact *in vivo.* The problem that many immunological studies have is capturing the complexity of multiple immunological parameters in an analytical framework.

Innate and adaptive immune responses are initiated sequentially in order to protect against different pathogens [4], although there is feedback and cooperation between the two systems. Innate γδ T cells are considered the first line of defence and development of adaptive naive CD4T cells often marks a secondary step if the innate immune system fails to cope with the infection [7]. Pro- (e.g-1 family, interleukin (IL)-6) and anti-inflammatory (transforming growth factor (TGF)-β) cytokines stimulate naive CD4T-cell differentiation into specific effector T-cell subsets or populations. The various Th1, Th2 and Th17 cytokines have the potential to induce, amplify or control innate immune cells, which aid in the clearance or containment of the pathogen. The three main groups of effector T cells tailor their function to the specific nature of the pathogen threat [8]: Th1 cells (e.g. interferon (IFN)-γ) are important for intracellular immunity against microorganisms; Th2 cells (e.g. IL-4, IL-13) respond to extracellular pathogens including helminths and protozoans; and Th17 cells (e.g. IL-17A, IL-17F, IL-22) tackle extracellular bacteria and fungi. Further cytokines (e.g. IL-10) are produced by regulatory T (Treg) cells which aim to restrain the pro-inflammatory nature of the Th response and minimize tissue damage. However, cytokines from one Th population have the potential to inhibit the production of a different Th subset. For example, IL-4 drives Th2 cell production and IL-10 but inhibits the production of IFN-γ [9], while the Th17-Treg interactions have been described as antagonistic and cooperative [10]. While there has been extensive study of the mechanisms and pathways involved for individual cytokines in host immune responses, there have been fewer studies of how these system components interact.

Understanding the response of the immune system is complicated because the individual cytokine responses are not independent but form a network of interacting responses. A great deal of past research on cytokine response to immunological challenge has used cross-sectional data and univariate or multivariate statistical techniques to investigate processes [11]. These do not take account of the interacting nature of the immune system nor do they consider the time-dependent nature of the responses. Structural equation modelling provides a framework by which the time-dependent nature of cytokine interactions can be analysed. A structural equation model (SEM) is an extension of pathway analysis and is subtly different to those cross-sectional and descriptive statistical frameworks in that it seeks to challenge a conceptual model of the pathways in the system based on prior knowledge. The relationships among cytokines are characterized by a series of equations that link multiple response variables to one or more predictors that are defined *a priori.* The goodness of fit of the conceptual pathway model to the data is assessed through analysis of the variance and covariance structure of the putative relationships in the network of pathways [12]. In this case, the equations are defined as hypothetical interactions between cytokines where individual cytokines may up- or downregulate each other. In complex pathways, the response from one equation can act as a predictor for other cytokine responses as defined further down the reaction pathway. A key feature of SEM is that pathways/networks are developed *a priori*, and it is therefore possible to challenge competing models of the system.

Structural equation modelling has been largely overlooked in analysing immunological processes [11]; perhaps because of the relatively small samples sizes that are frequently found in immunological studies. Small sample sizes (less than 100 or less than 10 times the number of connections between variables) are often considered inadequate for developing SEM using maximum-likelihood (ML) approaches [12]. In order to avoid such limitations, the SEM can be translated into a Bayesian framework [13,14], which has a number of advantages over the frequentist approach. The Bayesian approach incorporates prior knowledge about the parameters and does not rely on asymptotic theory and, therefore, has the potential to produce reliable results with small sample sizes [14,15].

Here, we show that the expression of a number of cytokines in response to *Campylobacter jejuni* challenge in commercial broiler chickens is breed-dependent and that the Th17 response emerges as predominant cytokine response using a combination of experiments and statistical modelling. The ever-increasing production requirement for uniform size and faster growth to harvestable size is believed to have had a negative impact on breed immunocompetence, especially to emerging zoonotic diseases [16]. We developed a conceptual model of cytokine interactions derived from published literature, which we challenged with experimental data from two commercial breeds using structural equation modelling to elucidate cytokine interactions in caecal tissue post-challenge. The primary site of *C. jejuni* colonization is the mucosal layer close to the epithelial cells in the deep crypts of the caecum at the terminal end of the gastrointestinal tract [17]. The relationship between *C. jejuni* and the chicken was originally thought to be commensal [18] with the inflammatory response being localized and self-limiting and not leading to severe pathology [19]. Recent investigations, however, indicate that the chicken–bacterium relationship cannot be considered as a commensal and severe mucosal damage can occur as a result of a prolonged pro-inflammatory response [20]. The expression of IL-1β and IL-6 had a positive impact on IL-17A emerging as the dominant interacting cytokines in what appears to be a protective response against *C. jejuni*. Th17 cells may act as important sentinels protecting mucosal surfaces in the gastrointestinal tract and as such will be upregulated as a result of the nature of immunological challenge presented by *C. jejuni* [17].

## 2. Material and methods

### 2.1. Bacterial strains and culture conditions

*Campylobacter jejuni* M1 was kindly provided by Dr Lisa Williams (University of Bristol). Bacteria were grown from stocks maintained at –80°C on Columbia blood agar (Lab M, Heywood, Lancashire, UK) supplemented with 5% defibrinated horse blood (Oxoid, Basingstoke, Hampshire, UK) for 48 h in microaerobic conditions (80% N_2_, 12% CO_2_, 5% O_2_ and 3% H_2_) at 41.5°C. Liquid cultures were grown for 24 h in 10 ml of Mueller-Hinton broth (MHB) in microaerobic conditions at 41.5°C and adjusted by dilution in fresh MHB to a final concentration of 106 CFU ml^−1^.

### 2.2. Animal husbandry and experimental design

The two broiler breeds used for the modelling study were housed in the University of Liverpool high-biosecurity poultry unit. Breed A reaches live slaughter weight (2.2 kg) at 36 days of age while breed B reaches a similar weight by 48 days. All animals were checked a minimum of twice daily to ensure their health and welfare; different breeds and control birds were housed separately; and all individuals were screened for *Campylobacter* using cloacal swabs prior to the experimental infection. Further details of animal husbandry can be found in Humphrey *et al.* [20].

Twenty-one days old broiler chicks, A (*n* = 40) and B (*n* = 40), were orally infected with 10^5^ cells of *C. jejuni* M1 in 0.2 ml of MHB. Control birds, A (*n* = 16) and B (*n* = 16) received 0.2 ml of sterile MHB. At 2, 5 and 12 days post-challenge, 10 infected and five control birds of each breed were chosen at random and killed by cervical dislocation. At post mortem examination, samples of tissue and gut contents were taken and processed for host gene expression analysis and *Campylobacter* enumeration.

### 2.3. Laboratory analysis

Caecal tissue samples from infected and control birds were collected and stored in 500 µl of RNAlater at −20°C (Sigma, Poole, Dorset, UK). Total RNA was isolated from 20 to 30 mg of tissue using an RNeasy minikit (Qiagen, West Sussex, UK) according to the manufacturer's instructions. Isolated RNA was eluted into 50 µl RNase-free water and stored at −80°C. The yield of total RNA was determined using a Nano-Drop (ND-1000) spectrophotometer. Expression of mRNA for the cytokines in caecal tissues was measured by real-time quantitative reverse transcription PCR (qRT-PCR) as previously described by Humphrey *et al.* [20]. Expression of the following additional cytokines were performed using our previously described methods; IL-13 [21], IL-19 [22], IL-17A (forward primer: CATGGGATTACAGGATCGATGA, reverse primer: GCGGCACTGGGCATCA, probe ACAACCGCTTCCCCCGCTTGG) and IL-17F (forward primer: TGACCCTGCCTCTAGGATGATC, reverse primer: GGGTCCTCATCGAGCCTGTA, probe: CAGGAATCGGTCTCTCGCTCCTTGG).

Expression of the target gene was determined using the cycle threshold (*C*_T_) value relative to that for the 28S rRNA reference gene (Δ*C*_T_). Results are expressed as fold changes in corrected target gene expression (Δ*C*_T_) in infected animals relative to the control animals (2^−ΔΔCT^).

### 2.4. Data analysis

The time-dependent expression of cytokines and subsequent modelling was undertaken in the R statistical programme (v. 3.0.3 [23]). Cytokine fold change was log(*x* + 1) transformed prior to analysis. Generalized linear models using quadratic, linear and log(time) relationships were used to examine the relationship between cytokine fold change and time. The Akaike information criteria with the lowest score was used to select the best-fitting model. Parameter values were then estimated using the R package FME [24] by fitting the best model to the data with upper and lower bounds and, secondly, by performing a separate Markov chain Monte Carlo (MCMC) simulation. In total, 100 000 iterations were used in the MCMC estimation process, with a burn-in of 10 000 and a thinning of 10. The MCMC simulation allowed estimates of the parameter confidence intervals.

A conceptual model was developed from published experiments that brought together knowledge on potential cytokine interactions. The studies included both whole organism and cultured cells from a variety of vertebrate hosts, including humans, mice and chickens. The conceptual model and whether bird genotype has any influence on the immune response was then challenged using an SEM. The current study generated a small sample size (*n* = 58). In order to avoid sample size limitations, we initially generated an SEM using a frequentist approach in order to describe the underlying processes within these data before translating this into a Bayesian framework [13,14]. The frequentist SEM (SEMf) was fit using ML estimation in the lavaan R-package [25]. The two genotypes used in this study have different growth performances, with genotype A gaining a larger maximum size than B. A Gompertz curve is the most appropriate nonlinear regression model for describing chicken growth curves [26]. It was fitted to the commercial breeds' growth performance data and the asymptotic value was used as an indication of maximum bird mass (kg) for the model. The growth curves were fitted using the FME R-package and parameter estimates are presented in the electronic supplementary material, table S1 and figure S1). The fold change values calculated for the cytokine data showed great variability and were log(*x* + 1) transformed prior to SEMf analysis. In addition, IL-17F was rescaled prior to log(*x* + 1) transformation by dividing all values by 10 because fold changes of more than 600 were recorded. Model adjustment and selection was initiated by removing non-significant parameters and by assessing ‘goodness of fit’, which was assessed using: (i) a *χ*^2^-test, where *p* > 0.05 indicates that the observed and expected covariance matrices for the model are not different; (ii) the root mean square approximation (RMSEA), with the lower confidence interval (CI) < 0.05 and upper 90% CI < 0.1; (iii) standardized root mean square residual (SRMR) < 0.08; and (iv) comparative fit index (CFI) > 0.95 [12,27,28].

The Bayesian SEM (SEMb) analysis was performed in JAGS interfaced with R using the rjags package [29] in order to test the significance and reliability of the SEMf parameter estimates and also to investigate whether small sample sizes could be analysed effectively in this framework JAGS use MCMC simulation based on the Gibbs sampling algorithm to generate a posterior distribution of the model parameters. Non-informative priors were used as there was little prior information [13]. In total, 200 000 iterations were used in the MCMC estimation process, with a burn-in of 100 000 and a thinning of 20. Model convergence was tested using the Gelman–Rubin convergence diagnostics which measures the difference within several chains and the variance between chains of the MCMC simulation by the potential scale reduction factor (psrf) [30,31]. If convergence is achieved then the MCMC chains should be indistinguishable and the Gelman–Rubin convergence diagnostic should return a multivariate psrf of 1. The SEMb parameter estimates were deemed significant when their 95% credible intervals excluded zero.

## 3. Results

### 3.1. Response of individual cytokines to *Campylobacter jejuni* challenge

**Figure 1.**
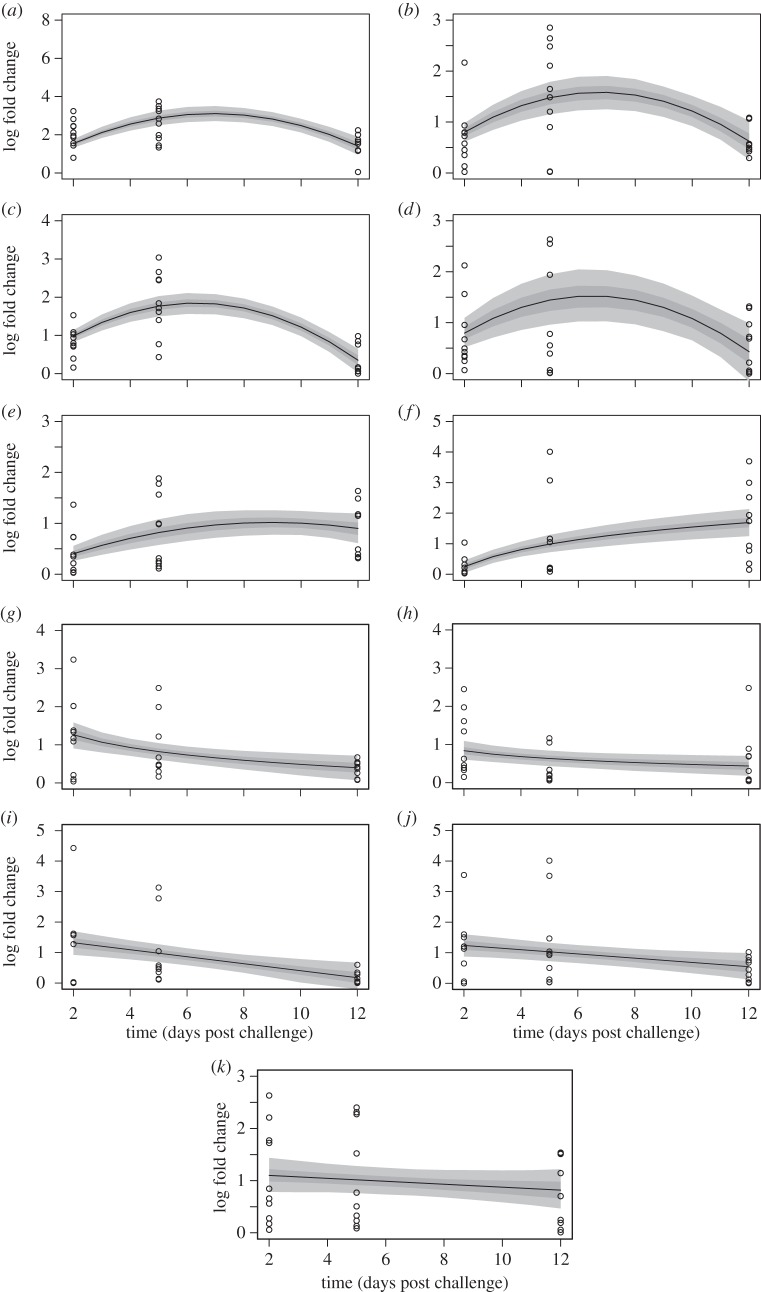
Time-dependent cytokine response post *Campylobacter jejuni* challenge in breed A (*a*) CXCLi2, (*b*) IL-1β, (*c*) TGF-β4, (*d*) IL10, (*e*) IFN-γ, (*f*) IL-17F, (*g*) IL-4 (*h*) IL-19, (*i*) IL-17A, (*j*) IL-6, (*k*) IL-13. The predictive envelope indicates the 25–75% (dark grey) and 5–95% (light grey) quantiles generated from the MCMC analysis of the parameter estimates.

**Figure 2.**
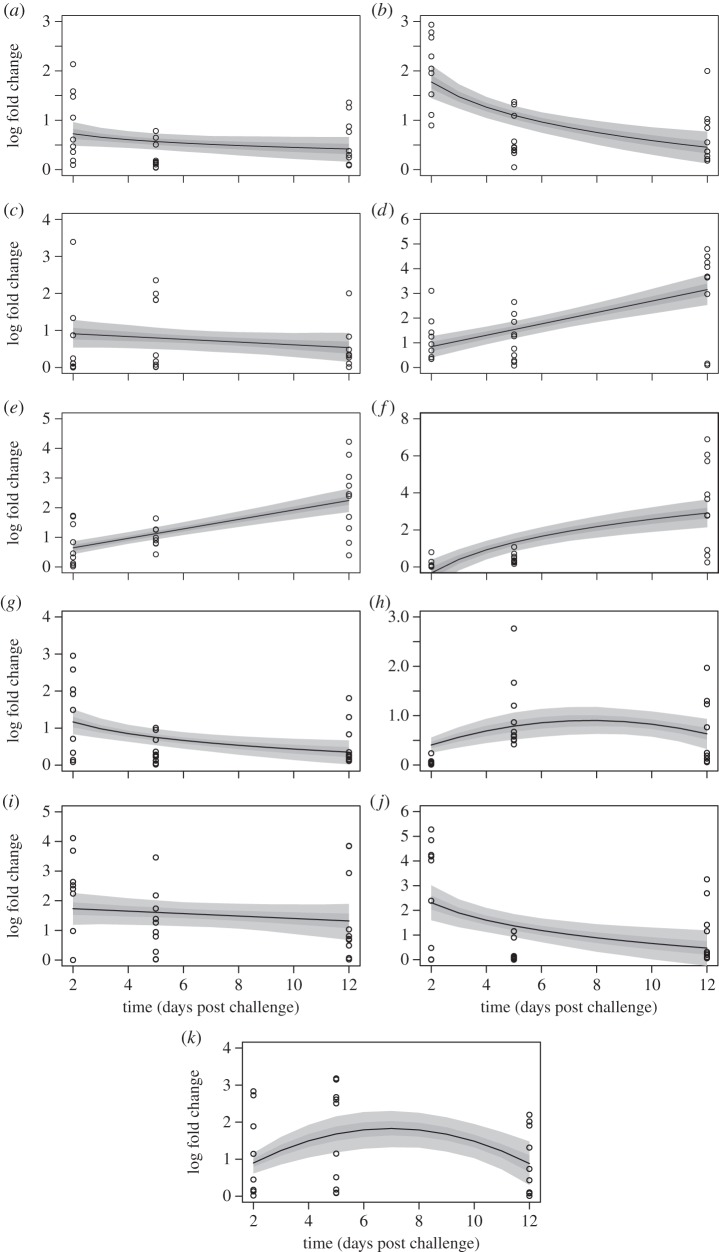
Time-dependent cytokine response post *Campylobacter jejuni* challenge in breed B (*a*) CXCLi2, (*b*) IL-1β, (*c*) TGF-β4, (*d*) IL-10, (*e*) IFN-γ, (*f*) IL-17F, (*g*) IL-4 (*h*) IL-19, (*i*) IL-17A, (*j*) IL-6, (*k*) IL-13. The predictive envelope indicates the 25–75% (dark grey) and 5–95% (light grey) quantiles generated from the MCMC analysis of the parameter estimates.

*Campylobacter jejuni* challenge elicited an immune response in the two breeds. In breed A, all he cytokines examined were upregulated but their responses varied over the duration of the experiment (figure 1). CXCLi2, IL-1β, TGF-β4, IL-10 and IFN-γ were initially upregulated but began to decrease over time while IL-17F continued to increase over the duration of the experiment (figure 1*a–f* and table 1). IL-4, IL-17A, IL-6, IL-19 and IL-13 all decreased from 2 to 12 days post-challenge but the relationship was only significant in IL-4 and IL-17A (figure 1*g–k* and table 1). Similar to breed A, upregulation of all the cytokines was observed in breed B (figure 2 and table 2). However, cytokine response over time varied between the two breeds. IL-10, IFN-γ and IL-17F all showed sustained increases over the experimental duration (figure 2*d–f* and table 2), while IL-19 response was greatest mid-way through (figure 2*h*). Parameter estimation indicated that CXCLi2, TGF-β4, IL-4, IL-17A, IL-6 and IL-13 all decreased from 2 to 12 days post-challenge (figure 2*b,c,g,i,j* and table 2) but the relationship was only significant for IL-4 and IL-6 (table 2).

**Table 1.** Parameter estimates and upper and lower 95% quantiles using Markov chain Monte Carlo (MCMC) simulation for the individual cytokine responses to *Campylobacter jejuni* challenge in breed A.

parameter	estimate (s.d.)	*p*-value	MCMC estimate (s.d.)	lower 95% quantile	upper 95% quantile
CXCLi2 = *a* × time^2^ + *b* × time					
*a*	−0.065 (0.038)	<0.001	−0.065 (0.0069)	−0.077	−0.054
*b*	0.90 (0.42)	<0.001	0.90 (0.076)	0.78	1.02
IFN-γ = *a* × time^2^ + *b* × time					
*a*	−0.012 (0.025)	<0.05	−0.012 (0.0048)	−0.020	−0.0044
*b*	0.22 (0.28)	<0.001	0.22 (0.053)	0.13	0.31
IL1-β = *a* × time^2^ + *b* × time					
*a*	−0.034 (0.032)	<0.001	−0.034 (0.0061)	−0.044	−0.024
*b*	0.46 (0.36)	<0.001	0.47 (0.067)	0.36	0.58
IL-10 = *a* × time^2^ + *b* × time					
*a*	−0.028 (0.040)	<0.001	−0.028 (0.0071)	−0.039	−0.016
*b*	0.39 (0.082)	<0.001	0.38 (0.079)	0.25	0.52
TGF-β4 = *a* × time^2^ + *b* × time					
*a*	−0.046 (0.027)	<0.001	−0.046 (0.0051)	−0.054	−0.037
*b*	0.58 (0.29)	<0.001	0.58 (0.056)	0.48	0.67
IL-4 = *a* + *b* × log(time)					
*a*	1.60 (1.78)	<0.001	1.61 (0.29)	1.11	2.13
*b*	−0.48 (1.03)	<0.05	−0.49 (0.16)	−0.76	−0.20
IL-17F = *a* + *b* × log(time)					
*a*	−0.30 (2.52)	0.51	−0.30 (0.16)	−0.55	−0.038
*b*	0.80 (1.46)	<0.05	0.80 (0.13)	0.59	1.04
IL-19 = *a* + *b* × log(time)					
*a*	0.99 (1.67)	<0.01	0.99 (0.26)	0.65	1.32
*b*	−0.22 (0.96)	0.22	−0.22 (0.10)	−0.39	−0.047
IL-6 = *a* + *b* × time					
*a*	1.38 (1.88)	<0.001	1.38 (0.26)	0.94	1.82
*b*	−0.07 (0.25)	0.14	−0.071 (0.031)	−0.12	−0.019
IL-17A = *a* + *b* × time					
*a*	1.56 (1.77)	<0.001	1.55 (0.28)	1.10	2.01
*b*	−0.11 (0.24)	<0.05	−0.11 (0.038)	−0.17	−0.05
IL-13 = *a* + *b* × time					
*a*	1.18 (1.52)	<0.001	1.15 (0.24)	0.76	1.54
*b*	−0.033 (0.20)	0.38	−0.027 (0.030)	−0.076	0.024

**Table 2.** Parameter estimates and upper and lower 95% quantiles using Markov chain Monte Carlo (MCMC) simulation for the individual cytokine responses to *Campylobacter jejuni* challenge in breed B.

parameter	estimate (s.d.)	*p*-value	MCMC estimate (s.d.)	lower 95% quantile	upper 95% quantile
CXCLi2 = *a* + *b* × log(time)					
*a*	0.84 (1.33)	<0.01	0.83 (0.21)	0.46	1.16
*b*	−0.17 (0.75)	0.22	−0.16 (0.12)	−0.36	0.04
IFN-γ = *a* + *b* × time					
*a*	0.32 (1.55)	0.27	0.32 (0.16)	0.058	0.59
*b*	0.16 (0.20)	<0.001	0.15 (0.026)	0.11	0.20
IL1-β = *a* + *b* × log(time)					
*a*	2.29 (1.64)	<0.001	2.28 (0.30)	1.81	2.80
*b*	−0.74 (0.92)	<0.001	−0.74 (0.17)	−1.02	−0.46
IL-10 = *a* + *b* × time					
*a*	0.37 (2.34)	0.44	0.38 (0.33)	−0.15	0.94
*b*	0.23 (0.30)	<0.001	0.22 (0.045)	0.15	0.30
TGF-β4 = *a* + *b* × time					
*a*	0.98 (1.76)	<0.01	0.97 (0.26)	0.53	1.40
*b*	−0.038 (0.22)	0.37	−0.037 (0.032)	−0.090	0.017
IL-4 = *a* + *b* × log(time)					
*a*	1.47 (1.86)	<0.001	1.47 (0.31)	0.94	2.00
*b*	−0.44 (1.04)	<0.05	−0.44 (0.17)	−0.73	−0.15
IL-17F = *a* + *b* × log(time)					
*a*	−1.56 (3.75)	<0.05	−1.55 (0.67)	−2.66	−0.44
*b*	1.80 (2.10)	<0.001	1.79 (0.37)	1.17	2.41
IL-19 = *a* × time^2^ + *b* × time					
*a*	−0.015 (0.029)	<0.01	−0.015 (0.0050)	−0.024	−0.0073
*b*	0.23 (0.32)	<0.001	0.23 (0.056)	0.14	0.33
IL-6 = *a* + *b* × log(time)					
*a*	3.00 (3.84)	<0.001	2.96 (0.65)	1.93	4.06
*b*	−1.02 (2.16)	<0.05	−1.00 (0.36)	−1.61	−0.38
IL-17A = *a* + *b* × time					
*a*	1.83 (2.52)	<0.001	1.82 (0.39)	1.17	2.48
*b*	−0.042 (0.32)	0.49	−0.041 (0.049)	−0.12	0.038
IL-13 = *a* × time^2^ + *b* × time					
*a*	−0.037 (0.052)	<0.001	−0.037 (0.0089)	−0.051	−0.022
*b*	0.52 (0.58)	<0.001	0.52 (0.10)	0.35	0.68

### 3.2. Development of a conceptual model to challenge with a structural equation model

**Figure 3.**
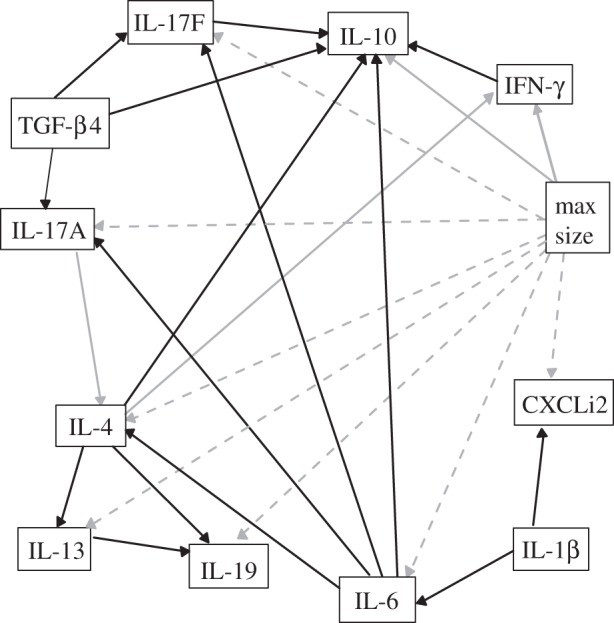
Full model depicting the potential response of cytokines and their interactions developed from peer-reviewed literature after challenge by a bacterial pathogen. This network of interactions is challenged by structural equation modelling. The arrows indicate the order of cytokine regulations: solid black arrows indicate a positive response or upregulation; sold grey arrows indicate a negative response or downregulation; and grey dashed arrows indicate that there is a negative relationship between maximum body size, used as a surrogate for breed type and cytokine response.

Examining the interaction of cytokines in response to *C. jejuni* challenge in chickens using SEM required the development of a conceptual *a priori* model from the published literature (figure 3). When a host recognizes specific bacterial ligands and microbial products an immune response is activated. An initial response produces the interleukins, IL-1β and IL-6 [19,32–34]. IL-1β plays a major role in the innate immune response and induces the production IL-6 and CXCLi2 [17,19,35,36]. The pattern of signals received by the host during the interaction with the pathogen will then determine whether a Th1, Th2 or Th17 response will be initiated [32]. The Th2 response is mediated through the upregulation of IL-4 [3,32,33], which can be stimulated by the production of IL-6 [37,38]. Production of IL-4 results in the induction of IL-13 and IL-19 [3,33,39]. IL-4 counteracts the IFN-γ function by suppressing the inflammatory response [40]. Th17 response is initiated by IL-1β, IL-6 and TGF-β can upregulate IL-17A and IL17F [41] and is negatively related to IL-4 and IFN-γ [42,43]. IL-10 production can be stimulated by cytokines of the Th1, Th2 and Th17 pathways [32,41,44] but its primary role is part of the Treg pathway of which TGF-β is the most important stimulating cytokine. This corresponds to a series of interactions as shown in figure 3.

### 3.3. Structural equation model of the cytokines response to *Campylobacter jejuni* challenge

The experiment provided data to investigate the cytokine interactions and assess which were involved in the hypothesized regulatory pathways detailed in the *a priori* model (figure 3). Model validation was initiated using the SEMf. The full *a priori* model was not an adequate representation of these data because of the non-significant parameter coefficients within the pathways and based on the RMSEA score (0.07) and the upper bounds of the 90% CI (0.12). IL-13 and IL-19, as well as a series of interaction pathways between these and other cytokines, including IFN-γ regulating IL-10 and a number of the cytokine-maximum size relationships, were removed from the model because they were non-significant. IL-4 (*p* = 0.056) and TGF-β4 (*p* = 0.057) influence on IL-10 and IL-17A, respectively, were marginally non-significant and therefore remained within the model.

**Figure 4.**
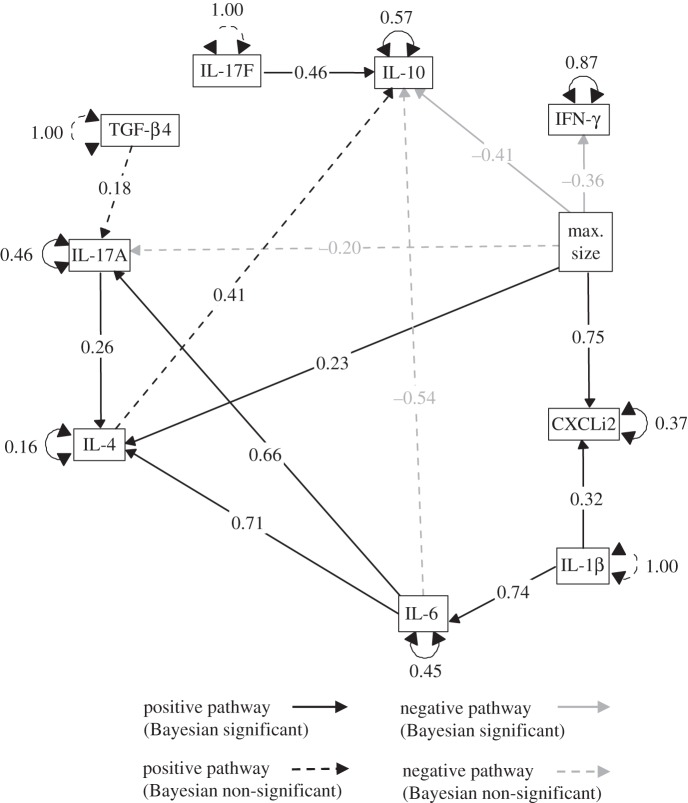
The final path model describing the cytokine interactions post-challenge in two breeds of broiler chicken. Positive relationships are indicated by black arrows, whereas grey arrows indicate a negative response. The standardized parameter estimates for each response are shown on each arrow. The unexplained variation for each of the variables, internally predicated by the model, is shown adjacent to their respective boxes.

The model adjustments greatly improved the ‘goodness of fit’. The final SEMf converged normally after 53 iterations: χ_22_^2^=19.30, *p* = 0.62; RMSEA = 0.00, 90% CI = 0.00–0.09; SRMR = 0.06; CFI = 1.00. The final SEMf including all the direct and indirect modelled cytokine interaction pathways is shown in figure 4 with the standardized coefficients while the unstandardized coefficients can be found in table 3. IL-17A response to TGF-β4 and IL-10 response to IL-4 did not improve in the final model (table 3).

**Table 3.** Coefficients, credible intervals and significance for the parameter estimates of the final frequentist structural equation model (SEMf) and Bayesian structural equation model (SEMb) for cytokine interactions in response to *Campylobacter jejuni* challenge in chickens. (The SEMf parameter estimates are unstandardized. The SEMb significant parameter estimates are highlighted in bold.)

response	predictor	SEMf parameter estimate (standard error)	*p*-value	SEMb parameter estimate	95% credible intervals
IL-10	IL17F	0.66 (0.14)	<0.001	**0.68**	**0.26–1.10**
	IL-4	0.67 (0.36)	0.06	0.64	−0.45 to 1.73
	IL-6	−0.51 (0.21)	<0.05	−0.50	−1.12 to 0.11
	max. size	−2.34 (0.60)	<0.001	−**2.16**	−**3.95 to -0.36**
IFN-γ	max. size	−1.37 (0.46)	<0.01	−**1.37**	−**2.62 to** −**0.14**
IL-4	IL-6	0.41 (0.04)	<0.001	**0.41**	**0.22–0.60**
	IL-17A	0.16 (0.04)	<0.001	**0.16**	−**0.05 to 0.38**
	max. size	0.77 (0.18)	<0.001	**0.77**	−**0.08 to 1.64**
IL-6	IL-1β	1.28 (0.15)	<0.001	**1.28**	**0.87–1.68**
CXCLi2	IL-1β	0.40 (0.10)	<0.001	**0.41**	**0–0.81**
	max. size	3.20 (0.34)	<0.001	**3.21**	**1.85–4.57**
IL-17A	IL-6	0.57 (0.08)	<0.001	**0.57**	**0.33–0.81**
	TGF-β4	0.25 (0.13)	0.05	0.25	−0.13 to 0.63
	max. size	−1.02 (0.49)	<0.05	−1.16	−2.61 to 0.26

The final SEMf was translated into a Bayesian framework. The SEMb was tested for convergence and whether it explained the observed variance in these data. The Gelman–Rubin convergence diagnostic test resulted in a multivariate psrf = 1. This can be visualized with the development of the psrf over the chain iterations (electronic supplementary material, figure S2). The MCMC chains all converged for the parameters and the model explained the variance observed in these data. The parameter estimates for the SEMf and SEMb were very similar (table 3) suggesting that the two modelling approaches were providing comparable results. The SEMb, however, indicated that 4 out of 14 parameter estimates were non-significant. The SEMb non-significant pathways were the relationships between: IL-10 and IL-4, IL-17A and TGF-β4 and the negative relationships between IL-10 and IL-6 and maximum body size and IL-17A (figure 4). Yet these pathways can still be considered marginally non-significant (table 3).

The exogenous variable maximum body size, a surrogate for bird breed, had a major impact on the cytokine response throughout the network. While there was no effect of breed on the cytokines associated with the initial cytokine response (IL-1β and IL-6), there were other effects deeper in the network. Breed type had an effect on CXCLi2 response to IL-1β, which can be interpreted as having an impact on a secondary step within a pathway (figure 4); i.e. upregulation of IL-1β by a pattern recognition receptor is step one while the response of CXCLi2 to IL-1β is step two. Breed type also had an effect on the third step of a pathway which impacted on IL-4, IL-17A and IL-10 responding to IL-6 (figure 4). There was a positive pathway that linked IL-1β to IL-4 and IL17A via IL-6 (figure 4). IL-1β was a positive driver of IL-6 and CXCLi2. IL-6 had a direct effect on IL-4 but also indirectly via IL-17A. IL-10 was influenced by IL-4, IL-6 and IL-17F, with the strongest relationship being between IL-10 and IL-6 which was negative.

## 4. Discussion

The primary aim of many immunological studies is to investigate changes in some form of immune status at a cellular, tissue or organ level over time in response to variations in the internal environment whether that is elicited by a pathogen, another agent or from an auto-immune perspective. The time-dependent analysis presented here, while based on a relatively small dataset, provided valuable information on which cytokines were up- or downregulated post *C. jejuni* challenge. However, it does not demonstrate cytokine interdependencies which leave knowledge of the system rather fragmented. Undertaking a structural equation modelling approach allowed us to investigate interactions among cytokines which allowed us to identify the most important pathways. We believe that the combined modelling approach provided a deeper understanding of the system because it identified which cytokines were upregulated and then allowed us to challenge a conceptual model of the theoretical relationships among cytokines with real data.

The time-dependent response and SEM indicated that there were differences between the faster, larger growing breed A and the slower growing, smaller breed B in their cytokine responses to *C. jejuni* challenge. Both breeds initiated an innate response through the upregulation of CXCLi2, IL-1β and IL-6. The SEM indicated that the level of CXCLi2 expression was breed-dependent, as there was a positive relationship between the exogenous variable maximum size and expression of CXCLi2, although expression of IL-1β and IL-6 were not related to maximum size. CXCLi2 is homologous with human IL-8 and is important for an early immune response in the gastrointestinal tract, especially for its chemotactic role in monocyte and heterophil recruitment [3,45]. The upregulation of CXCLi2 suggested that breed A and B initiated a functional innate response to *C. jejnui* [20]. However, the subsequent upregulation of IFN-γ, IL-17A and IL-10 and their levels expressed through time, along with CXCLi2, indicated the course of the immunological response to *C. jejuni* varied between the two breeds. The SEM identified this through a negative relationship between size and IFN-γ, IL-17A and IL-10. Both IFN-γ and IL-17A are important effector T cells and play a crucial role in the clearance of bacterial pathogens [8,19,32]; they are also potent pro-inflammatory mediators. The anti-inflammatory IL-10 is often expressed by T regulatory cells and is important in restraining the pro-inflammatory response. The differential response of IFN-γ, IL-17A and IL-10 has implications for how the two breeds fight infection but also how each breed controls inflammation. We have previously shown that the inflammatory response between these breeds differs, with prolonged inflammation and inflammatory damage occurring in the faster growing breed A, even though invasion and colonization are similar [20].

The Th17 response was clearly identified in the final SEMf and was in agreement with the SEMb along with the upregulation of innate immune system chemokine CXCLi2. In mammals, the Th17 pathway responds to extracellular bacterial infection and while frequently associated with inflammatory conditions, also plays a key role as a sentinel response in the intestinal tract, preventing invasion by bacterial pathogens and maintaining gut integrity [4]. While the role of the Th17 pathway has yet to be fully elucidated in the chicken, it is likely to play a similar role particularly during *C. jejuni* infection which colonizes the host in the mucosal layer of the deep crypts within the caecum. The chemotactic properties of IL-17 make it an important inducer of CXCLi1 and CXCLi2 chemokines. Yet, the SEM did not identify any IL-17-CXCLi2 association within the model's covariance structure. The key cytokines resulting in the induction of IL-17A expressed within the SEM is largely in agreement with that expected from experimental studies [46] but the SEM did not explain all of the variation observed in IL-17A nor did it implicate any cytokines in the upregulation of IL-17F. The SEM indicated that IL-17A production potentially started with IL-1β which had a positive effect on IL-6 and in turn stimulated IL-17A. IL-1β combined with IL-6, a STAT3 activator, are important for Th17 cell differentiation. IL-1β has a crucial role at the initial stages of the Th17 cell differentiation because it upregulates the expression of IL-1 receptors and retinoic acid receptor-related orphan receptors-γt on the surface of Th17 lymphocytes resulting in the subsequent production of IL-6 and IL-17A [47].

The SEM also indicated that approximately 50% of IL-17A variance was unexplained. The cytokine cascade involving IL-1β, IL-6 and IL-17A potentially relates to the differentiation of naive CD4T cells which are part of the adaptive immune response. The unexplained variation will undoubtedly be related to interactions with other cytokines or induction pathways that were not captured within the model. IL-23 was not part of the suit of cytokines examined post-experimentation but it has a role in activating and sustaining Th17 cell development [7], leading to the production of IL-17 cytokines that induce localized tissue inflammation [48]. Toll-like receptors (TLR) on innate immune γδ T cells can produce IL-17A [49]. TLR are a group of receptors that are involved in orchestrating the innate immune response and recognize bacteria and microbial products. *Campylobacter jejuni* activates TLR2 in chickens [50], which in mouse models is believed to interact directly with pathogens resulting in the upregulation of IL-17 [49]. Intestinal epithelium harbour important populations of γδ T lymphocytes where they act as a first line of defence against pathogens [51]. γδ T cells are more numerous in the chicken accounting for up to 60% of the peripheral lymphocytes and are numerous in mucosal sites such as the gastrointestinal and reproductive tracts [51–53]. We hypothesize that γδ T cells may play a key role in avian mucosal immunity producing a substantial amount of IL-17.

There was only a weak relationship between IL-17A and TGF-β4 while TGF-β4 appeared to have no effect on IL-17F, IL-10 or IFN-γ. TGF-β is often implicated in conjunction with IL-6 as important for the upregulation of IL-17 cytokines even though Th17 cytokines can differentiate in a TGF-β independent manner [46]. The role of the anti-inflammatory TGF-β in regulating Th17 cell differentiation is complex because there appears to be differences in the relative importance of TGF-β in the Th17 response depending on the system being studied [46,48]. In humans, there is evidence for TGF-β-dependent and -independent [46,54] production of IL-17 in conjunction with other cytokines but in mice TGF-β is a requirement for successful development of Th17 cells [46]. Human and mouse model systems focus on TGF-β1 but TGF-β4 in chickens is believed to have the same functional role [3]. The role of TGF-β *in vivo* is likely to be dependent on a number of environmental stimuli not to mention interactions with other cytokines, which may not be evident when particular genes or cytokines are experimentally suppressed. The weak relationship observed in the SEM may indicate the TGF-β4 is not involved in the dominant IL-17 producing pathway or that TGF-β4 concentrations are below a particular threshold, which in conjunction with IL-6 and IL-1β, allows the production of IL-17A instead of Treg [55,56].

Both IL-10 and TGF-β are anti-inflammatory cytokines which have the potential to suppress Th1, Th2 and Th17 responses. IL-10 is crucial for the control of inflammation during the host's response to a pathogen and can be regulated by the co-induction of Th1 [44], TGF-β [57] and IL-4. Neither IFN-γ nor TGF-β appeared to have an effect on IL-10 in the SEM, either directly or indirectly via examination of the model's covariance structure. The SEM indicated that IL-6 had a negative effect on IL-10. IL-6 production has the potential to block Treg activity. In mammals, IL-6 induction through STAT3 phosphorylation downregulates FOXP3 and blocks Treg cell differentiation while maintaining the Th17 response in order to continue dealing with the infection [58,59]. Although our understanding of chicken Tregs is rudimentary a similar process may be happening here. The fact that IL-6 has a negative effect on IL-10 and there is no relationship between IL-10 and IFN-γ or TGF-β4 provided more evidence that the chicken's response is aimed at fighting an extracellular bacterial infection. However, both IL-4 and IL-17F had a positive effect on IL-10, suggesting these cytokines were interacting in a positive manner. It may be that the IL-6 positive effect on IL-4 in turn allows the upregulation of IL-10 through a different pathway. Such an interaction would potentially allow the anti-inflammatory properties of IL-10 to control tissue inflammation and protect the host. However, without further experimental studies this assertion is speculative.

The results are of biological as well as analytical interest because the approach provided an opportunity to validate the potential for using an SEM to investigate cytokine interactions [11]. From an infection immunology perspective, these modelling approaches can add new insight into the complex interactions of cytokine responses in a system such as the gut. While we realize that we have analysed only part of the immune system response to challenge with a pathogen, there is no reason why this analytical approach could not be expanded to include other key components of the immune system beyond cytokines, e.g. γδ T cell or naive CD4T cell proliferation, or measures of inflammation as a latent variable. Structural equation modelling may even assist in identifying which immunological variables result in recurrence of disease [60]. It is clear that these statistical techniques have considerable potential in defining the essential protective responses or crucial differences in responses that underlie host immune responses to any form of infection or challenge to the immune system.

In this study, the overarching hypothesis was that bird genotype impacts on the immunocompetence of commercial broiler chickens and that this would be evident in the cytokine response. We used a combined modelling approach that first assessed the time-dependent cytokine response of two breeds of broiler chicken to *C. jejuni* challenge before examining cytokine interactions in a generalized multi-equation framework facilitated by structural equation modelling [61]. Both SEM approaches demonstrated that bird genotype had an effect on cytokine interactions post *C. jejuni* challenge in caecal tissue as well as identifying interdependences among cytokines, which have the potential to be cytokine cascades. The fact that IL-13 and IL-19 were removed from the final model and IFN-γ appeared to be only related to bird genotype potentially indicated that the Th17 response was the prominent cytokine pathway observed. The prominence of the Th17 pathway suggests that broiler chickens' immune response was directed at controlling an extracellular bacterial pathogen through restricting it to the gut lumen.

## Supplementary Material

Cytokine responses in birds challenged with the human food-borne pathogen Campylobacter jejuni implies a Th17 response The file contains extra information about the model parameters and diagnostics for the Bayesian structural equation model.

## Data accessibility

The data used in this study are available from the Dryad Digital Repository and can be found under the following: http://dx.doi.org/10.5061/dryad.7h3v1.
