# Supplementary material for: Cytokine responses in birds challenged with the human food-borne pathogen Campylobacter jejuni implies a Th17 response
Source: R Soc Open Sci. 2016 Mar 16;3(3):150541. doi: 10.1098/rsos.150541 (PMC4821255; doi:10.1098/rsos.150541)
Supplement: Cytokine responses in birds challenged with the human food-borne pathogen Campylobacter jejuni implies a Th17 response The file contains extra information about the model parameters and diagnostics for the Bayesian structural equation model. [file rsos150541supp1.docx]

**Cytokine responses in birds challenged with the human food-borne pathogen *Campylobacter jejuni* implies a Th17 response**

William D. K. Reid^1*^, Andrew J. Close^1^, Suzanne Humphrey^2^, Gemma Chaloner^2^, Lizeth Lacharme-Lora ^2^, Lisa Rothwell^3^, Pete Kaiser^3^, Nicola J. Williams^4^, Tom J. Humphrey^5^, Paul Wigley^2^ and Steven P. Rushton^1^

*corresponding author; email: [william.reid@newcastle.ac.uk](mailto:william.reid@newcastle.ac.uk); phone: +44 191 208 3076

**Supplementary material**

Breed growth performance

The breeder’s growth performance data were used to calculate growth rates (r) and maximum body mass (K) in kilograms using a Gompertz growth curve (equation 1). The breed growth performance objectives are supplied by companies that develop chicken breeds for commercial use give the expected growth rate under good management and environmental conditions with the correct balance of nutrients within the diet. The parameters r and K, therefore, reflect genotypic differences between the two breeds of broiler chickens used within this study.

The breed growth performance data were inversed modelled using the FME package in R version 3.0.3 in order to find r and K. Inverse modelling allows the data to be fit to a model and is often the most practical way to find parameter estimates. The collinear nature of r and K mean that they can be used interchangeably.

The Gompertz growth curve used to the model to these data was;

dM/dt <- r*log(K/M)*M (equation 1)

where M is the initial body mass (kg).

The parameter estimates for the two breeds are in Supplementary table 1.

Supplementary Table 1: Growth parameter estimates for breeds of commercial broiler chicken.

| Breed | Parameter | Estimate | t-value | p-value |
| --- | --- | --- | --- | --- |
| Breed A | r (growth rate) | 0.043852 | 203.0 | <2e-16 |
|  | K (maximum size) | 6.186292 | 162.5 | <2e-16 |
| Breed B | r (growth rate) | 0.041378 | 102.41 | 2.23e-12 |
|  | K (maximum size) | 3.444862 | 79.23 | 1.34e-11 |

Supplementary Figure 1 illustrates the difference between the two breeds.

Supplementary Figure 1: The difference in growth performance between two commercial broiler chicken breeds.

Supplementary Figure 2: Gelman-Rubin plots showing the development of the potential scale reduction factor (psrf) for modelled parameters over the 200 000 iterations and 4 chains. Individual plot titles represent the modelled relationship for which the parameter is being estimated. Multivariate psrf = 1.
